# Noninvasive evaluation of the hemodynamic status in patients after heart transplantation or left ventricular assist device implantation

**DOI:** 10.1371/journal.pone.0275977

**Published:** 2022-10-14

**Authors:** Sebastian Roth, Henrik Fox, René M’Pembele, Michiel Morshuis, Giovanna Lurati Buse, Markus W. Hollmann, Ragnar Huhn, Thomas Bitter

**Affiliations:** 1 Department of Anesthesiology, University Hospital Duesseldorf, Duesseldorf, Germany; 2 Clinic for Thoracic and Cardiovascular Surgery, Herz- und Diabeteszentrum NRW, Ruhr Universität Bochum, Bad Oeynhausen, Germany; 3 Heart Failure Department, Herz- und Diabeteszentrum NRW, Ruhr-Universität Bochum, Bad Oeynhausen, Germany; 4 Department of Anesthesiology, Amsterdam University Medical Center (AUMC), Amsterdam, The Netherlands; 5 Department of Anesthesiology, Kerckhoff Heart and Lung Center, Bad Nauheim, Germany; 6 Department of Pneumology and Respiratory Medicine, Staedtisches Klinikum Braunschweig, Braunschweig, Germany; University of Messina, ITALY

## Abstract

**Introduction:**

Hemodynamic assessment is crucial after heart transplantation (HTX) or left ventricular assist device (LVAD) implantation. Gold-standard is invasive assessment via thermodilution (TD). Noninvasive pulse contour analysis (NPCA) is a new technology that is supposed to determine hemodynamics completely noninvasive. We aimed to validate this technology in HTX and LVAD patients and conducted a prospective single-center cohort study.

**Methods:**

Patients after HTX or LVAD implantation underwent right heart catheterization including TD. NPCA using the CNAP Monitor (V.5.2.14; CNSystems Medizintechnik AG, Graz, Austria) was performed simultaneously. Three TD measurements were compared with simultaneous NPCA measurements for hemodynamic assessment. To describe the agreement between TD and NPCA, Bland–Altman analysis was done.

**Results:**

In total, 28 patients were prospectively enrolled (HTX: n = 10, LVAD: n = 18). Bland-Altman analysis revealed a mean bias of +1.05 l/min (limits of agreement ± 4.09 l/min, percentage error 62.1%) for cardiac output (CO). In LVAD patients, no adequate NPCA signal could be obtained. In 5 patients (27.8%), any NPCA signal could be detected, but was considered as low signal quality.

**Conclusion:**

In conclusion, according to our limited data in a small cohort of HTX and LVAD patients, NPCA using the CNAP Monitor seems not to be suitable for noninvasive evaluation of the hemodynamic status.

## Introduction

After heart transplantation (HTX) or left ventricular assist device (LVAD) implantation, hemodynamic assessment plays a crucial role [1]. Hemodynamic assessment is not only important in the acute postoperative phase, but also in follow-up visits to evaluate graft function or LVAD functionality [2,3]. Current gold-standard is invasive assessment via thermodilution (TD) using a Swan-Ganz-catheter [4]. However, this method is invasive and may be associated with certain risks such as injuries to the nerves and vessels, cardiac arrhythmias or infections [5–7]. Noninvasive pulse contour analysis (NPCA) is a promising new technology that is supposed to determine hemodynamics completely noninvasive via two simple finger cuffs [8]. These inflatable cuffs keep the capillary blood flow constant. The pressure needed is recorded by a pressure transducer and corresponds to the true arterial pressure waveform (= vascular unloading technique or so-called Penaz principle) [9]. Based on the arterial pressure waveform (= pulse contour), it is then possible to determine cardiac output as the area under the arterial curve correlates with cardiac stroke volume. Further hemodynamic parameters such as cardiac index (CI) and systemic vascular resistance (SVR) can be calculated on this basis.

Several studies investigated the accuracy and precision of hemodynamic assessment using NPCA in diverse cohorts and revealed conflicting results [10–13]. In patients post HTX or LVAD implantation, no data are available yet. However, NPCA may be a promising technology in these cohorts as all of these patients regularly need an evaluation of their hemodynamic status and NPCA may provide an estimation of cardiac output (CO) without risks. In the following, this may help to avoid invasive assessment, especially when patients are clinically stable. E.g., patients might receive noninvasive assessment in advance to get an idea if invasive assessment is necessary or not. Therefore, we aimed to investigate if NPCA is a suitable method for noninvasive hemodynamic assessment in patients after HTX or LVAD implantation.

## Methods

### Study design

A prospective single-center cohort study was conducted. The study was approved by the Ethical Review Board of the Ruhr University Bochum (Registration number: 47/2016) and all patients gave written informed consent. The study was performed in compliance with the declaration of Helsinki and according to the guidelines for good clinical practice (GCP). This report follows the STARD guidelines.

### Participants

Patients after HTX or LVAD implantation were prospectively enrolled. All patients were clinically stable and had left the intensive care unit at the time of investigation. Exclusion criteria were age < 18 years, severe tricuspid valve dysfunction and a noninvasive blood pressure (NIBP) difference ≥ 20 mmHg between left and right arm before investigation. Patients with severe tricuspid valve dysfunction were excluded as this might lead to an overestimation of CO by TD. Patients with blood pressure difference were excluded as this might result in wrong blood pressure calibration with consecutive wrong measurements. All participating patients have been recruited parallelly to another clinical trial that investigated NPCA in patients with chronic heart failure and has been published previously [11].

### Test methods

Patients underwent right heart catheterization including TD which was routinely performed during patients’ hospitalization in an outpatient department. At least three TD-CO measurements were performed using 10 ml boluses of cold saline (< 10 degree Celsius). Cold saline was randomly injected throughout the respiratory cycle as performed previously [4,11]. Single measurements were excluded if variability exceeded 10% compared with mean results. For data analysis, the mean value of three TD-CO measurements within a range of ≤ 10% was used. CI, SV and SVR were calculated. Auto calibrated NPCA was performed simultaneously. Patients were connected with the CNAP Monitor (V.5.2.14; CNSystems Medizintechnik AG, Graz, Austria) via two finger cuffs. An oscillometric blood pressure cuff was used for calibration measurement. CO was recorded continuously on a beat-to-beat basis. Documentation of NPCA values was started at the time of cold saline injection. In HTX patients, TD measurements were only allowed if the NPCA signal was visually considered adequate. In LVAD patients, no physiological arterial pressure waveform with adequate signal quality could be expected. Therefore, hemodynamic measurements were also performed if any NPCA signal with the possibility to calculate hemodynamic parameters was available. The mean value of recorded beat-to-beat NPCA measurements was averaged and used for data analysis.

### Analysis

All statistical analyses were performed using IBM SPSS version 26. Continuous variables are presented as means with standard deviation or as median with interquartile range, as appropriate. Categorical variables are presented as counts and percentages. To describe the agreement between TD and NPCA, Bland–Altman analysis was done. This statistical method assesses the mean difference (bias) and calculates the limits of agreement (LOA = ± 1.96 x SD of bias of the methods) to find out the variance of the values and is recommended as the gold standard to compare two methods for CO measurement [8].

## Results

### Participants

**Fig 1:**
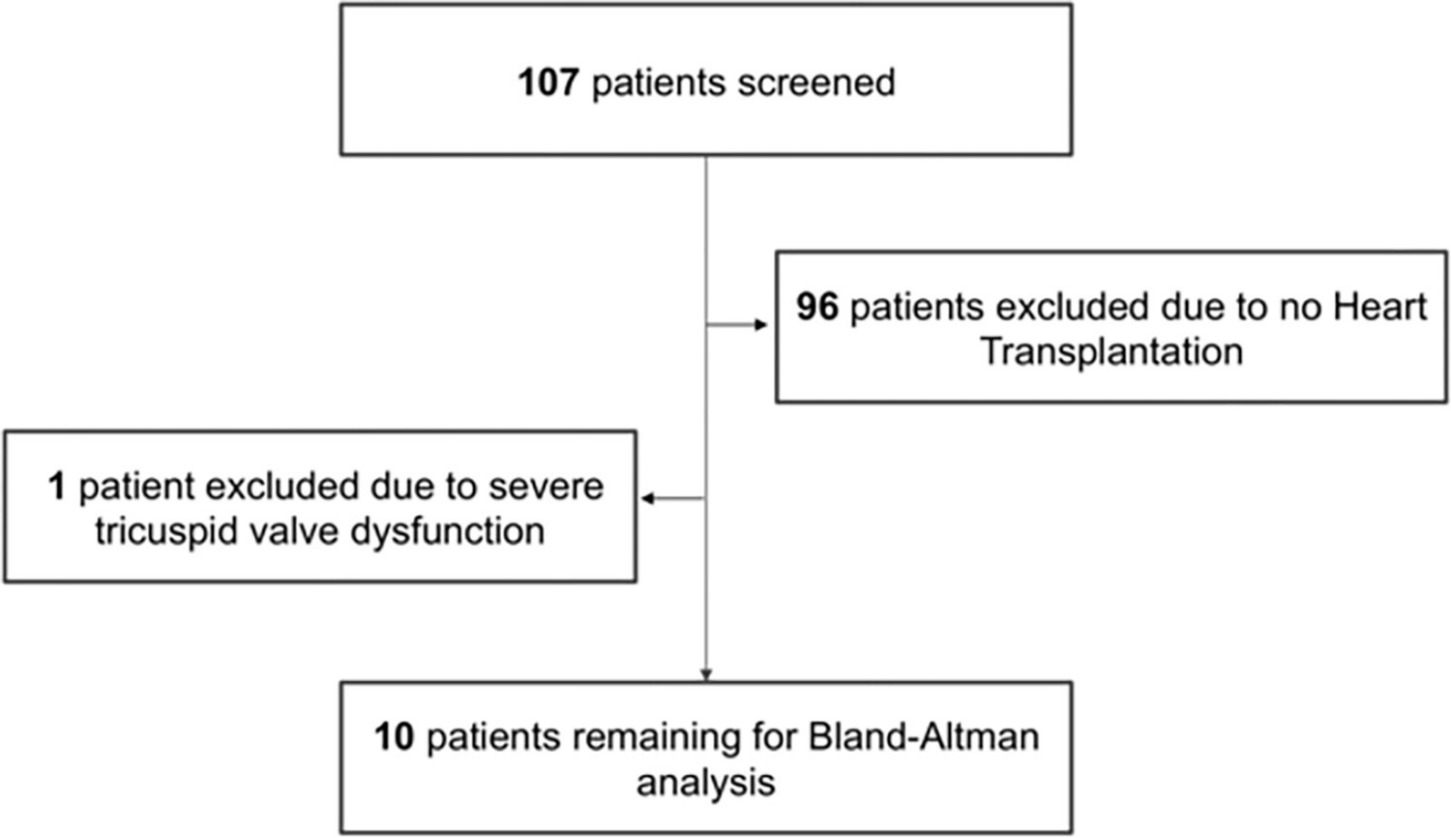
Flow chart.

In total, 107 patients were screened for this trial. 29 patients met the inclusion criteria for the enrollment of HTX and LVAD patients. One patient had to be excluded due to severe tricuspid valve dysfunction. Finally, 28 prospectively enrolled patients (10 HTX patients (90% male, age 47 ± 8 years, LVEF 61 ± 7%) and 18 LVAD patients (LVEF 24 ± 5%, 89% male, age 53 ± 10 years, pulsatile pump n = 4; continuous-flow pump n = 14)) could be included into the study (Fig 1). Detailed patient characteristics can be found in Table 1.

**Table 1:** Patient characteristics HTX and LVAD patients.

HTX patients	n	%	mean	SD
**Baseline data**				
Age			47	±8
Male sex	9	90		
BMI			27.3	±3.5
LVEF (%)			61	±7
LVEDD (mm)			47	±5
LAD (mm)			43	±6
Severe heart valve regurgitation / stenosis	0	0		
Sinus Rhythm	10	100		
BNP (pg/ml)			297	±319
Troponin I (pg/ml)			44	±66
Hemoglobin (g/dl)			11.9	±2.5
Creatinine (mg/dl)			1.86	±1.22
Glomerular filtration rate (ml)			49	±21
History of chronic kidney disease	7	70		
History of diabetes mellitus	0	0		
**Medication**				
ACE inhibitors	4	40		
Angiotensin receptor blocker	2	20		
Diuretics	8	80		
Beta-blocker	4	40		
Digitalis	0	0		
Amiodarone	0	0		
**Hemodynamic data**				
Heart rate (bpm)			90	±15
Mean arterial pressure (mmHg)			108	±11
Central venous pressure (mmHg)			10	±5
Mean pulmonary arterial pressure (mmHg)			23	±7
Cardiac output (l/min)			6.1	±1.5
Cardiac index (l/min/m2)			2.9	±0.9
Stroke volume (ml)			70	±22
Systemic vascular resistance (dynsec/cm5)			1397	±451
**LVAD patients**	**n**	**%**	**mean**	**SD**
**Baseline data**				
Age			53	±10
Male sex	16	89		
BMI			28.5	±6.5
LVEF (%)			24	±5
LVEDD (mm)			62	±17
LAD (mm)			47	±9
Severe heart valve regurgitation / stenosis	3	16.7		
Ischemic heart disease	11	61.1		
Dilative cardiomyopathy	7	38.9		
Diabetes mellitus	4	22.2		
BNP (pg/ml)			935	±1112
Hemoglobin (g/dl)			11.6	±2.8
Creatinine (mg/dl)			1.94	±1.13
Glomerular filtration rate (ml)			47	±23
History of chronic kidney disease	12	66.7		
History of diabetes mellitus	4	22.2		
**Medication**				
ACE inhibitors	7	38.9		
Angiotensin receptor blocker	2	11.1		
Diuretics	15	83.3		
Beta-blocker	17	94.4		
Digitalis	3	16.7		
Amiodarone	8	44.4		
LVAD with pulsatile pump	4	22.2		
LVAD with continous flow pump	14	77.8		
Estimated pump speed (rpm)			7670	±1360
**Hemodynamic data**				
Heart rate (bpm)			75	±15
Mean arterial pressure (mmHg)			80	±18
Central venous pressure (mmHg)			11	±8
Mean pulmonary arterial pressure (mmHg)			24	±10
Cardiac output (l/min)			4.5	±0.8
Cardiac index (l/min/m2)			2.2	±0.5
Stroke volume (ml)			69	±11
Systemic vascular resistance (dynsec/cm5)			1249	472

Data are presented as absolute values with corresponding percentages or as mean values ± standard deviation. SD = Standard Deviation; BMI = Body Mass Index; LVEF = Left Ventricular Ejection Fraction; LVEDD = Left Ventricular Enddiastolic Diameter; LAD = Left Atrial Diameter; BNP = Brain Natriuretic Peptide; ACE = Angiotensin-Converting Enzyme; LVAD = Left Ventricular Assist Device.

### Test results

#### HTX patients

**Fig 2:**
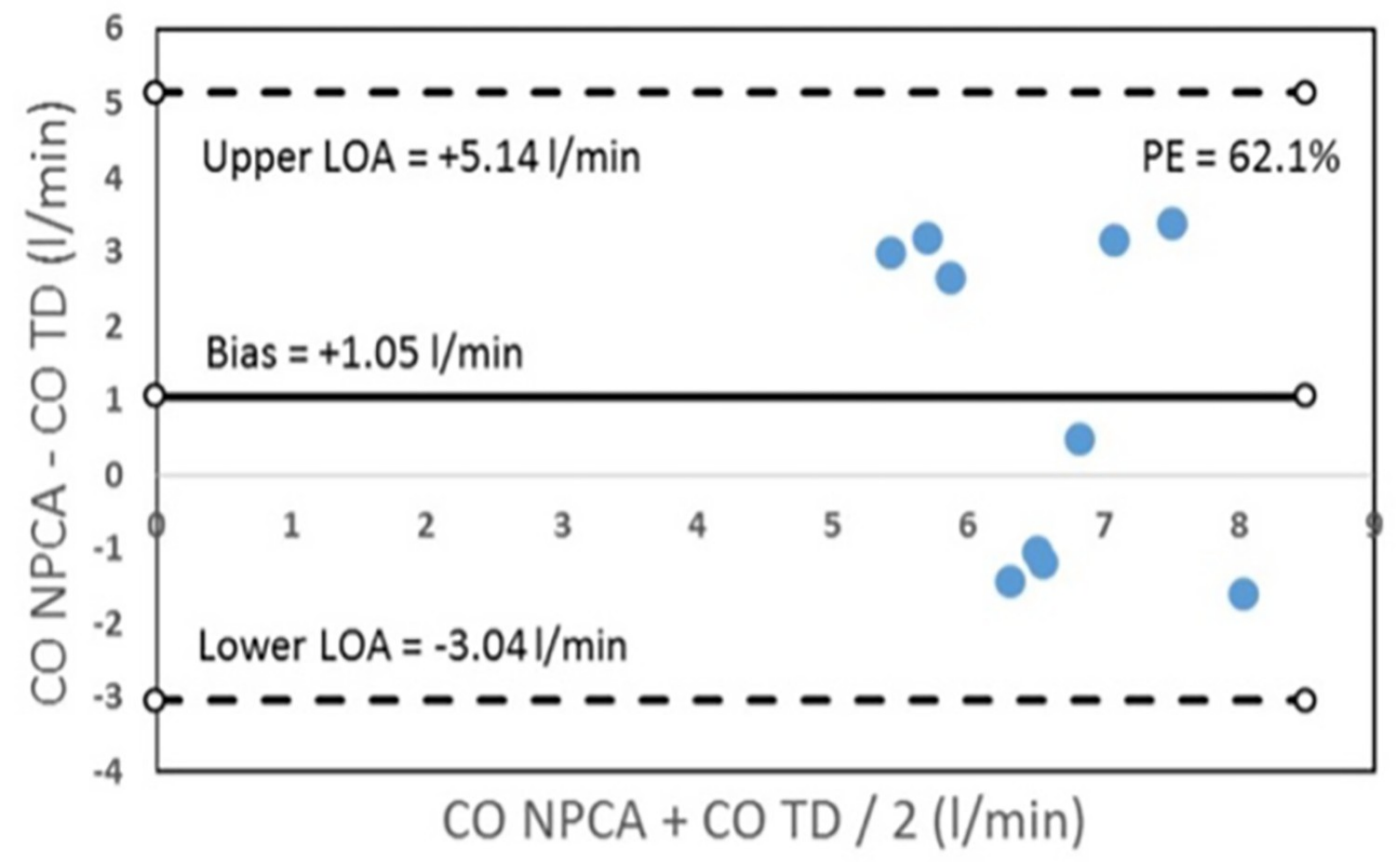
Bland-Altman plot for cardiac output (CO); Bias, limits of agreement (LOA) and percentage error (PE) were calculated.

In HTX patients, hemodynamic assessment using TD or NPCA using the CNAP Monitor demonstrated a significant difference for mean CO (TD 6.06 ± 1.48 l/min, NPCA 7.12 ± 1.08 l/min, p<0.001), CI (TD 2.92 ± 0.86 l/min/m^2^, NPCA 3.36 ± 0.42 l/min/m^2^, p<0.001), SV (TD 70 ± 22 ml, NPCA 86 ± 17 ml, p<0.001), and SVR (TD 1397 ± 451 dyne*s*cm^-5^, NPCA 1108 ± 189 dyne*s*cm^-5^, p<0.001). Bland-Altman analysis revealed a mean bias of +1.05 l/min (limits of agreement (LOA) ± 4.09 l/min, percentage error (PE) 62.1%) for CO, +0.45 l/min/m^2^ (LOA ± 1.92 l/min/m^2^, PE 61.1%) for CI, +16 ml (LOA ± 49 ml, PE 63.1%) for SV, and -289 dyne*s*cm^-5^ (LOA ± 887 dyne*s*cm^-5^, PE 70.8%) for SVR (Table 2 and Fig 2).

**Table 2:** Hemodynamic assessment HTX patients–Thermodilution versus NPCA.

	TD	NPCA	Bias	LOA	PE (%)
**CO (l/min)**	6.06±1.48	7.12±1.08	1.05±2.09	±4.09	62.1
**CI (l/min/m** ^**2**^ **)**	2.92±0.86	3.36±0.42	0.45	±1.92	61.1
**SV (ml)**	70±22	86±17	16	±49	63.1
SVR (dyn*s*cm ^**-5**^ **)**	1397±451	1108±189	-289	±887	70.8

Data are presented as mean values ± standard deviation. TD = Thermodilution; NPCA = Noninvasive Pulse Contour Analysis; LOA = Limit of Agreement; PE = Percentage error; CO = Cardiac Output; CI = Cardiac Index; SV = Stroke Volume; SVR = Systemic Vascular Resistance.

### LVAD patients

In all 18 LVAD patients, no adequate NPCA signal by the CNAP Monitor could be obtained. In patients with pulsatile pumps (n = 4), NPCA signal with any arterial pressure curve could be detected so that noninvasive measurement of hemodynamics could be performed as values for analysis. In patients with continuous-flow pumps, only 1 patient had any NPCA signal that revealed hemodynamic parameters to be included into analysis. In the remaining 13 LVAD patients with continuous flow pumps, no NPCA signal with the possibility to calculate hemodynamic parameters could be detected. Accordingly, hemodynamic data of only 5 LVAD patients have been analysed. In these 5 patients, hemodynamic assessment using TD or NPCA demonstrated a significant difference for mean CO (TD 4.74 ± 0.74 l/min, NPCA 5.58 ± 1.61 l/min, p<0.001), CI (TD 2.34 ± 0.41 l/min/m^2^, NPCA 2.69 ± 0.62 l/min/m^2^, p<0.001), SV (TD 69 ± 9 ml, NPCA 77 ± 20 ml, p<0.001), and SVR (TD 1303 ± 216 dyne*s*cm^-5^, NPCA 1126 ± 692 dyne*s*cm^-5^, p<0.001). Due to the inacceptable signal quality and the limited number of only 5 patients, Bland-Altman analysis was not done in this sub-group to avoid overinterpretation of these data.

## Discussion

With regard to our results, we have to conclude that NPCA using the CNAP Monitor is not suitable to evaluate the hemodynamic status in patients after HTX or LVAD implantation. While in HTX patients the percentage error (= 62.1%; Fig 2) did not meet the statistical criterion standard by Critchley and Critchley (≤ 30%) [14], in LVAD patients, signal quality of NPCA by the CNAP Monitor was low and any NPCA signal could be detected in only 27.8% of patients. In the following, we want to discuss some possible reasons for the limited measurement performance of NPCA in these cohorts.

In HTX patients, two factors may have influenced the insufficient measurement performance of NPCA: 1) the patients’ characteristics and 2) the calibration mode [15]. Referring to HTX patients’ characteristics, one possible etiology might relate to the phenomenon of vasoplegia which can be observed regularly after HTX. With regard to Table 2, we can see that CO was overestimated and SVR was underestimated by NPCA. However, in our study, it is not very likely that relevant vasoplegia was still present at the time of investigation as all patients had left the intensive care unit and were clinically stable.

A further factor that might complicate the use of NPCA after HTX consists in the possibility of denervation. Transplantation of a new heart surgically interrupts the parasympathetic vagal neurons and the intrinsic postganglionic sympathetic nerves which causes extrinsic cardiac denervation [16]. In the following, this may lead to discrepancies between cardiac hemodynamics and the control of peripheral vasoconstriction / vasodilation.

Another aspect in terms of patients characteristics refers to the vascular status of HTX patients. One can assume that the accuracy of a completely noninvasive pulse contour analysis device suffers from poor vascular status which might be present after HTX, e.g. due to numerous arterial catheterizations for invasive blood pressure measurement or because of severe arteriosclerosis. Although this assumption seems plausible from a clinical perspective, evidence is also lacking.

The second main factor next to patient characteristics that may have influenced the results of NPCA refers to the calibration mode. It is important to mention that NPCA calculated CO on the basis of biometric patient data in this study. A recent study by our working group revealed that, in comparison to the current gold standard TD, auto-calibrated NPCA using the CNAP Monitor systematically overestimates CO with decrease in cardiac function in patients with severe chronic heart failure (NYHA-class III-IV) [11]. Moreover, with decreasing CI as determined by TD, there was an increasing gap between CO values obtained by TD and NPCA (*r* = − 0.75, *p* < 0.001). These data suggest that NPCA may not be able to detect low CO values at all. In the present study, left ventricular function according to echocardiopraphy was good (mean LVEF = 61%±7) and CI was also in the normal range. Nevertheless, this aspect should be considered when interpreting absolute NPCA values in patients after HTX. Against this background, one of the main messages of this manuscript is that although NPCA seems to perform well in healthy subjects, this technology might have problems in patients who really need evaluation of hemodynamic status such as patients after HTX or LVAD implantation or patients with severe chronic heart failure.

On the other hand, there are also studies that could reveal more promising results: Wagner et al. have not only assessed the agreement between absolute CNAP Monitor-based CO values, but also performed a trend analysis. They could show in 51 intensive care unit patients after cardiothoracic surgery that NPCA is able to track changes of CO due to passive leg raising maneuvers with an accordance rate of 100% in four-quadrant plots (exclusion zone 0.5 l/min) [10]. Unfortunately, we have not performed any trend analysis to measure CO changes over time, which is a major limitation of this study (see limitations section). Future studies should address this aspect in more detail.

In LVAD patients, our results show clearly that this population seems not to be suitable for noninvasive hemodynamic assessment via NPCA as in only 27.8% of patients, any NPCA signal could be obtained by the CNAP Monitor. In this sub-group, hemodynamic assessment also revealed significantly different values in comparison with the gold standard TD. Most patients (4/5, 80%) with any NPCA signal had LVADs with pulsatile pumps. In this context, it is important to mention that pulsatile pumps are no longer utilized today. Unfortunately, we cannot say how many of the continuous-flow LVAD patients had aortic valve opening (and therefore some degree of arterial pulsatility) at the time of NPCA evaluation. This information would be helpful to denote if the NPCA signal was unable to be obtained as there was no pulsatility or because the degree of pulsatility was so low that the signal was inadequate. In contrast, patients exhibiting myocardial recovery while on LVAD who exhibit aortic valve opening with every beat and significant pulsatility on arterial waveform might prove to be a subpopulation of LVAD patients in whom NPCA might prove helpful.

An additional aspect specifically for LVAD patients refers to the interference between LVAD pumps and NPCA technologies. Possibly, technological factors such as speed or magnetic levitation of the LVAD pump may lead to different measurement results by NPCA. We cannot provide evidence on this assumption, but we think that this point should also be considered when evaluating NPCA in LVAD patients.

Regarding the current literature, studies investigating NPCA in LVAD patients are missing. However, some studies investigated invasive pulse contour analysis, for example the study by Scoletta et al. [17] In this study, a good correlation was found between TD-CO and invasive pulse contour analysis so that the authors concluded that this method may be a complementary tool in the hemodynamic assessment of patients supported with LVAD. The discrepancies between this study and our results might be explained by the fact that invasive assessment performs more accurate than noninvasive assessment. Referring to studies that investigated noninvasive pulse wave analysis devices in general, there is a meta-analysis by Saugel et al. which concluded that study heterogeneity in the literatur12e is high and the pooled results revealed that CO measurements were not interchangeable in surgical or critically ill patients [18].

### Limitations

This study has several limitations: First, the sample size of 28 patients is small so that it is not possible to draw final conclusions. It is definitely necessary to perform further prospective studies in HTX and LVAD patients in larger cohorts. Also, as the nature of this study was exploratory, no formal sample size calculation has been performed. Second, a major limitation of this study is that we did not perform any trend analysis. Even if measurement of absolute values is inaccurate, measurement of trends might help to evaluate hemodynamic status in the course of time, e.g. to see if a new therapeutic approach has been successful or not. Third, we cannot provide any information on the presence of aortic valve opening in LVAD patients as these data were not included into our database. It would definitely be interesting to investigate if this factor correlates with the quality of NPCA signal. Fourth, no patient had invasive blood pressure monitoring at the time of investigation so that we cannot provide information if blood pressure measurement using NPCA was accurate in this study. For LVAD patients, blood pressure measurement via doppler was also not available. Fifth, in HTX patients, we cannot report data on the transplanted organs which may also be a factor influencing hemodynamics. Sixth, some baseline characteristics in this study are not representative (e.g. history of diabetes mellitus in 0/10 HTX patients). This may also be related to the very limited sample size. Seventh, and finally, the direct Fick method might be more suitable to measure CO in LVAD patients compared to TD. However, necessary values for mixed venous oxygen saturation and oxygen consumption were not available so that Fick method-based CO could not be calculated.

### Conclusions

In conclusion, according to our data, NPCA using the CNAP Monitor seems not to be suitable to evaluate hemodynamic status in patients after HTX or LVAD implantation. Therefore, NPCA by the CNAP Monitor cannot be recommended for this purpose so far. As the sample size in this study was small, it is not possible to draw final conclusions. Nevertheless, our data add to the limited literature in this field and might be helpful for clinicians to correctly interpret hemodynamic parameters by NPCA in HTX and LVAD patients. This is important as clinical decisions might be based on these values. Further studies are needed to clarify if correction factors may improve accuracy of NCPA or if this technology might not be able at all to evaluate hemodynamics noninvasively in patients who really need it, such as patients with HTX or LVAD.

## References

[1] Ponikowski P, Voors AA, Anker SD, Bueno H, Cleland JGF, Coats AJS, et al. 2016 ESC Guidelines for the diagnosis and treatment of acute and chronic heart failure. Eur Heart J. 2016;37: 2129–2200m. doi: 10.1093/eurheartj/ehw128 27206819

[2] Schramm R, Zittermann A, Morshuis M, Schoenbrodt M, von Roessing E, von Dossow V, et al. Comparing short-term outcome after implantation of the HeartWare® HVAD® and the Abbott® HeartMate 3®. ESC Hear Fail. 2020;7: 908–914. doi: 10.1002/ehf2.12649 32190985 PMC7261579

[3] Gummert JF, Haverich A, Schmitto JD, Potapov E, Schramm R, Falk V. Permanent implantable cardiac support systems. Dtsch Arztebl Int. 2019;116: 843–848. doi: 10.3238/arztebl.2019.0843 31931951 PMC6970315

[4] Ganz W, Donoso R, Marcus HS, Forrester JS, Swan HJC. A new technique for measurement of cardiac output by thermodilution in man. Am J Cardiol. 1971;27: 392–396. doi: 10.1016/0002-9149(71)90436-x 4929422

[5] Iberti T, Benjamin E, Gruppi L, Raskin J. Ventricular arrhythmias during pulmonary artery catheterization in the intensive care unit prospective study. Am J Med. 1985;78: 451–454. 3976703 10.1016/0002-9343(85)90337-7

[6] Szymczyk T, Sauzet O, Paluszkiewicz LJ, Costard-Jäckle A, Potratz M, Rudolph V, et al. Non-invasive assessment of central venous pressure in heart failure: a systematic prospective comparison of echocardiography and Swan-Ganz catheter. Int J Cardiovasc Imaging. 2020;36: 1821–1829. doi: 10.1007/s10554-020-01889-3 32445006 PMC7497509

[7] Bossert T, Gummert JF, Bittner HB, Barten M, Walther T, Falk V, et al. Swan-Ganz catheter-induced severe complications in cardiac surgery: Right ventricular perforation, knotting, and rupture of a pulmonary artery. J Card Surg. 2006;21: 292–295. doi: 10.1111/j.1540-8191.2006.00235.x 16684066

[8] Saugel B, Cecconi M, Wagner JY, Reuter DA. Noninvasive continuous cardiac output monitoring in perioperative and intensive care medicine. Br J Anaesth. 2015;114: 562–575. doi: 10.1093/bja/aeu447 25596280

[9] Molhoek GP, Wesseling KH, Settels JJM, van Vollenhoven E, Weeda HWH, de Wit B, et al. Evaluation of the Penàz servo-plethysmo-manometer for the continuous, non-invasive measurement of finger blood pressure. Basic Res Cardiol. 1984;79: 598–609. doi: 10.1007/BF01910489 6508716

[10] Wagner JY, Körner A, Schulte-Uentrop L, Kubik M, Reichenspurner H, Kluge S, et al. A comparison of volume clamp method-based continuous noninvasive cardiac output (CNCO) measurement versus intermittent pulmonary artery thermodilution in postoperative cardiothoracic surgery patients. J Clin Monit Comput. 2018;32: 235–244. doi: 10.1007/s10877-017-0027-x 28540614

[11] Roth S, Fox H, Fuchs U, Schulz U, Costard-Jäckle A, Gummert JF, et al. Noninvasive pulse contour analysis for determination of cardiac output in patients with chronic heart failure. Clin Res Cardiol. 2018;107: 395–404. doi: 10.1007/s00392-017-1198-7 29352326

[12] Fischer MO, Avram R, Cârjaliu I, Massetti M, Gérard JL, Hanouz JL, et al. Non-invasive continuous arterial pressure and cardiac index monitoring with Nexfin after cardiac surgery. Br J Anaesth. 2012;109: 514–521. doi: 10.1093/bja/aes215 22750726

[13] Smetkin AA, Hussain A, Kuzkov V V., Bjertnæs LJ, Kirov MY. Validation of cardiac output monitoring based on uncalibrated pulse contour analysis vs transpulmonary thermodilution during off-pump coronary artery bypass grafting. Br J Anaesth. 2014;112: 1024–1031. doi: 10.1093/bja/aet489 24531685

[14] Critchley LAH, Critchley JAJH. A meta-analysis of studies using bias and precision statistics to compare cardiac output measurement techniques. J Clin Monit Comput. 1999;15: 85–91. doi: 10.1023/a:1009982611386 12578081

[15] Wagner JY, Grond J, Fortin J, Negulescu I, Schöfthaler M, Saugel B. Continuous noninvasive cardiac output determination using the CNAP system: evaluation of a cardiac output algorithm for the analysis of volume clamp method-derived pulse contour. J Clin Monit Comput. 2016;30: 487–493. doi: 10.1007/s10877-015-9744-1 26227161

[16] Awad M, Czer LSC, Hou M, Golshani SS, Goltche M, De Robertis M, et al. Early denervation and later reinnervation of the heart following cardiac transplantation: A review. J Am Heart Assoc. 2016;5: 1–21. doi: 10.1161/JAHA.116.004070 27802930 PMC5210323

[17] Scolletta S, Miraldi F, Romano SM, Muzzi L. Continuous cardiac output monitoring with an uncalibrated pulse contour method in patients supported with mechanical pulsatile assist device. Interact Cardiovasc Thorac Surg. 2011;13: 52–57. doi: 10.1510/icvts.2010.264234 21454314

[18] Saugel B, Hoppe P, Nicklas JY, Kouz K, Koerner A, Hempel JC, et al. Continous noninvasive pulse wave analysis using finger cuff technologies for arterial blood pressure and cardiac output monitoring in perioperative and intensive care medicine: a systematic review and meta-analysis. Br J Anaesth. 2020;125: 25–37. doi: 10.1016/j.bja.2020.03.013 32475686

